# Editorial: Psychological and cognitive evaluation and intervention based on physiological and behavioral computing

**DOI:** 10.3389/fncom.2023.1249197

**Published:** 2023-07-31

**Authors:** Lin Shu, Matthieu Perreira Da Silva, Lulu Zhang, Chee-Kong Chui

**Affiliations:** ^1^School of Future Technology, South China University of Technology, Guangzhou, Guangdong, China; ^2^Nantes Université, École Centrale Nantes, CNRS, LS2N, UMR 6004, Nantes, France; ^3^Guangzhou First People's Hospital, Guangzhou, Guangdong, China; ^4^Department of Mechanical Engineering, National University of Singapore, Singapore, Singapore

**Keywords:** psychological computing, virtual reality, artificial intelligence, cognitive evaluation, physiological signals, behavioral data

Physiological and behavioral computing is a tool for evaluating mental states and cognitive processes. However, traditional approaches to psychological and cognitive research using physiological and behavioral data have faced several challenges. With the advent of advanced technology, it is now possible to design more effective paradigms using virtual reality, gaming, and new scales, among others. Wearable and non-contact technologies make it possible to monitor multiple physiological and behavioral signals, facilitating the establishment of psychological and cognitive evaluation models with better generalization performance.

This Research Topic aims to summarize the foundations and technologies of physiological and behavioral computing for psychological and cognitive evaluation and intervention. It includes task design, cognitive assessment using virtual reality, gaming, language, and new scales, among others. Additionally, the topic covers the acquisition and processing of physiological and behavioral data, psychological calculation based on physiological signals, as well as cognitive evaluation methods with multimodal signal fusion.

The Research Topic presents eight articles. These papers showcase the potential of physiological and behavioral computing for psychological evaluation and intervention, highlighting the importance of combining information from multiple modalities to improve accuracy and effectiveness.

Chen et al. proposes a novel approach to emotion recognition using multi-modal physiological signals, and the results demonstrate the effectiveness of the proposed 1D convolutional neural network-based method compared to traditional machine learning approaches.

Chung examines the relationship between users' claustrophobic tendencies and their need for space in digital interfaces. The association of stronger claustrophobic tendencies toward physical space and stronger need for digital space was reported in two studies. Such tendencies are likely to influence cognitive tasks on digital devices more than device-related spatial needs. This study provides valuable insights on users' spatial needs in fast-changing digital environments.

Dai et al. investigates the effects on executive vigilance when transcranial direct current stimulation (tDCS) is applied over the left dorsolateral prefrontal cortex (DLPFC). The findings suggest that anodal tDCS over left DLPFC enhances the early stage of relevant information processing and the inhibitory control of distracting stimuli during continuous and monotonous vigilance task.

Fordson et al. proposes a deep learning model for classifying emotional responses based on electrodermal activity signals. The model incorporates knowledge graphs as a way to learn representations from the data, as well as gender and age information and time and frequency EDA features. The authors introduce a weighted feature fusion method to combine knowledge embedding feature vectors and statistical feature vectors for emotional state classification. The results show a significant improvement in the performance of the emotion recognition system, demonstrating the potential of physiological and behavioral computing for psychological evaluation and intervention.

The study in the paper (Vargas et al.) presents a novel use of virtual reality, machine learning and implicit measures for assessment of empathy. Participants were immersed in virtual environments representing social workplace scenarios while their eye-gaze patterns and behaviors were recorded. The findings suggest that the combination of eye-gaze patterns and behaviors during virtual reality experiences may differentiate between empathy dimensions.

The relationship between trait anxiety and attentional bias is unclear. Xing et al. explored the impact of trait anxiety on attentional bias toward emotional stimuli using materials from the International Affective Picture System. Participants with high trait anxiety showed greater avoidance of threatening stimuli during the early period of stimulus presentation. They also displayed a stronger attentional bias toward positive and dysphoric stimuli during the middle and late periods. Hence, trait anxiety may influence the temporal aspects of attention to emotional stimuli, providing insights into the relationship between trait anxiety and selective attention.

Zhang, Yang et al., presents an automatic schizophrenia detection algorithm based on the reading deficit of schizophrenic patients using multimodality media. The proposed algorithm utilizes abnormal speech, head movement, and reading fluency during the reading task to detect schizophrenia. The results obtained indicate that the proposed approach is efficient enough to be used for the assisted auxiliary diagnosis of schizophrenia.

Zhang, Yin et al. proposes a real-time deep learning framework to detect acute stress by fusing multimodal information, including ECG, voice, and facial expressions. The framework utilizes ResNet50 and I3D with the temporal attention module to extract stress-related information, and the matrix eigenvector-based approach to fuse multimodality information. The effectiveness of the framework was validated using the Montreal imaging stress task (MIST) experiment. This work demonstrates the potential of using deep learning technology to detect stress and the importance of combining information from multiple modalities.

In conclusion, this Research Topic presents a collection of papers that demonstrate the potential of physiological and behavioral computing for psychological evaluation and intervention. The results highlight that a thoughtful integration of information from multiple modalities can enhance the accuracy and effectiveness of psychological and cognitive assessment and intervention. It is important to note that the development of new methods and technologies for psychological and cognitive research, as well as for the diagnosis and treatment of mental health disorders, does not follow a one-size-fits-all approach.

## Author contributions

All authors listed have made a substantial, direct, and intellectual contribution to the work and approved it for publication.

